# Effects of intermittent visual feedback on EEG characteristics during motor preparation and execution in a goal-directed task

**DOI:** 10.3389/fnhum.2024.1371476

**Published:** 2024-12-12

**Authors:** Baobao Yu, Yimeng You, Yahui Li, Jiaqi Chen, Huilin Zhou, Jun Wang, Junchen Huang, Weinv Fan, Jialin Xu, Guokun Zuo

**Affiliations:** ^1^Ningbo Institute of Materials Technology and Engineering, Chinese Academy of Sciences, Ningbo, Zhejiang, China; ^2^Ningbo Cixi Institute of Biomedical Engineering, Ningbo, Zhejiang, China; ^3^Ningbo No.2 Hospital, Ningbo, Zhejiang, China; ^4^University of Chinese Academy of Sciences, Beijing, China

**Keywords:** electroencephalography, intermittent visual feedback, goal-directed tasks, motor preparation, motor-related cortical potential, event-related desynchronization

## Abstract

**Introduction:**

Visual feedback plays a crucial role in goal-directed tasks, facilitating movement preparation and execution by allowing individuals to adjust and optimize their movements. Enhanced movement preparation and execution help to increase neural activity in the brain. However, our understanding of the neurophysiological mechanisms underlying different types of visual feedback during task preparation and execution remains limited. Therefore, our study aims to investigate the impact of different types of visual feedback on movement-related brain activity in goal-directed tasks, in order to identify more effective forms of visual feedback in goal-directed tasks.

**Methods:**

The electroencephalographic (EEG) data from 18 healthy subjects were collected under both continuous and intermittent visual feedback conditions during a goal-directed reaching task. We analyzed the EEG characteristics of the event-related potential (ERP), event-related synchronization/desynchronization (ERS/ERD) in all subjects during motor preparation and execution of the goal-directed reaching task.

**Results:**

The results showed that, the amplitude of motor-related cortical potential (MRCP) in subjects was larger in the intermittent visual feedback condition compared to the continuous visual feedback condition during motor preparation, and the amplitude was largest at the CPz electrode. Additionally, mu-ERD was more pronounced during both motor preparation and execution under intermittent visual feedback condition.

**Discussion:**

In conclusion, intermittent visual feedback enhanced the characteristics of subject’s brain activation and cortical excitability in the time and time-frequency domains.

## 1 Introduction

Goal-directed tasks, such as reaching movements, involves three main processes for their sensory control: the perception of movement, the generation of motor intentions involving preparatory neural activity within the brain, and the execution of movement (Erlhagen and Schöner, 2002). These tasks facilitate brain reorganization during the movement preparation and execution stages, inducing neural activity in the relevant brain regions (Grosse-Wentrup et al., 2011; Mrachacz-Kersting et al., 2012). Previous studies have demonstrated that goal-directed tasks can activate the posterior parietal cortex more extensively in subjects as they plan and prepare to perform the tasks (Pereira et al., 2017). Compared to non-targeted tasks, goal-directed tasks are more likely to promote movement preparation and help individuals have better movement execution outcomes (Frömer et al., 2012). Additionally, these tasks were found to elicit a larger amplitude of movement-related cortical potential (MRCP), indicating enhanced motor preparation and execution.

The MRCP is an event-related potential (ERP) used to study movement preparation and execution, reflecting changes in brain activity associated with movement over time. As an electroencephalogram (EEG) indicator of cortical excitability, larger amplitudes of MRCP represent increased synchrony of neural activity in motor areas of the brain, and may also represent increased neuronal firing and enhanced synaptic connections (Elbert and Rockstroh, 1987; Birbaumer et al., 1990). Bereitschaftspotential (BP) is the early component of the MRCP and primarily reflects the processing of movement preparation (Shakeel et al., 2015). “Early BP” occurs approximately 1,500 ms before the movement onset, and “late BP” develops about 400–500 ms before the movement onset, which has a maximum amplitude over the primary motor cortex (Shakeel et al., 2015). The amplitude of MRCP may relate to the amount of effort required for the movement, while the duration of MRCP is interpreted as the length of time taken to plan and prepare for the movement (Wright et al., 2011). The study by Skrzeba et al. showed that the greater the effort exerted by the subjects, the larger the amplitude of the MRCP (Grosse-Wentrup et al., 2011; Skrzeba and Vogt, 2018; Sanchez-Lopez et al., 2014).

In goal-directed tasks, visual input plays an important role when people prepare for and execute movements. Most of this visual input is in the form of goals, which provide individuals with critical information about the purpose and direction of the task (Hu et al., 2024). They use this information to accomplish the task. Visual feedback is a type of visual input, which is crucial for people to adjust and optimize their movements while performing a task (Shafer et al., 2019; Castronovo et al., 2019). Therefore, goal-related visual feedback has become a focus of research and attention. Wasaka et al. designed a goal-directed task with continuous visual feedback, and found that the MRCP amplitude of subjects under visual feedback condition increased compared to without visual feedback (Wasaka et al., 2022). This indicated that the addition of visual feedback in a goal-directed task increased neural activity in the cerebral cortex. In addition, other studies have also shown that motor tasks with visual feedback evoked higher magnitude of event-related desynchronization (ERD) compared to tasks without feedback (Wagner et al., 2014).

Event-related desynchronization/synchronization (ERD/ERS) represents brain activity changes related to movement in the time-frequency domains. ERD/ERS mainly involves cortical oscillations at mu (8–13 Hz) and beta (14–30 Hz) frequencies (Meirovitch et al., 2015). It has been proposed that the magnitude of the ERD in the mu and beta bands is influenced by levels of cortical excitability, with an increased amplitude of ERD indicating heightened cortical excitability (Kasuga et al., 2015). The magnitude of the ERD also reflects the neural activity involved in movement planning (Nakayashiki et al., 2021). The mu-ERD is enhanced when people prepare for and perform tasks (Li et al., 2018; Gu et al., 2009), and ERD of mu band becomes stronger in goal-directed tasks (Nakayashiki et al., 2021). The MRCP amplitude and ERD of the subjects were significantly enhanced in the goal-directed task with visual feedback (Trincado-Alonso et al., 2018).

Researchers also have studied characteristics of visual feedback, such as frequency. Some researchers have investigated the effects of different frequencies of visual feedback on motor performance, and the results have shown that higher frequencies visual feedback can enhance people’s performance during movement execution (Slifkin et al., 2000; Lafe et al., 2016a, 2016b). It has also been pointed out that the frequency of visual feedback needs to be reduced as the motor skill level of the subject improves. Because with the improvement of the subjects’ mastery of motor skills, they will rely more on muscle sensations to perform tasks, leading to a decrease in information obtained from the current movement and a decrease in initiative (Guadagnoli and Lee, 2004). At this stage, reducing the frequency of external visual feedback can encourage the subjects to rely more on internal sensory feedback, thus improving their motor performance and adaptability (Guadagnoli and Lee, 2004; Wulf and Shea, 2002; Wulf et al., 2001). Researchers typically consider a feedback frequency of 60 Hz as continuous visual feedback and have conducted studies on it. Sanford et al. conducted a comparative study of continuous and intermittent visual feedback, and the behavioral results indicated that intermittent visual feedback improved subjects’ motor performance (Sanford et al., 2022). Other researchers have found that intermittent visual feedback increased cortical excitability during motor execution compared to continuous visual feedback (kang et al., 2012).

However, our comprehension of the neurophysiological mechanisms that underlie various forms of visual feedback during motor preparation and execution is currently limited. Therefore, we have focused on designing different visual feedback to investigate brain activity during the motor preparation and execution in a goal-directed task for individuals. In this study, we compared continuous visual feedback with intermittent visual feedback and analyzed the EEG characteristics of subjects in the time domain and time-frequency domain. We hypothesized that the MRCP and mu-ERD would exhibit distinct patterns under the two types of visual feedback conditions, and that participants would need to exert greater effort to complete the task with intermittent visual feedback. We proposed a design for visual feedback that is more conducive to enhancing brain activity, allowing subjects to better prepare for movement and increase their brain activation. This may facilitate the improvement of motor function in people.

## 2 Materials and methods

### 2.1 Participants

Twenty healthy adults with normal or corrected visual acuity participated in the experiment. All subjects were right-handed and had no history of psychiatric or neurological disorders. Subjects were recruited from the Cixi Institute of Biomedical Engineering. EEG data from two subjects were excluded due to poor quality, such as severe drift, excessive muscle artifacts, or excessive eye movements, before data processing. EEG data from 18 subjects (10 males and 8 females, mean age of 23.8 ± 1 years) were ultimately retained. All experimental procedures were conducted in accordance with the Declaration of Helsinki, and all participants signed a written informed consent that was approved by the local Research Ethics Committee before the start of the experiment.

### 2.2 Data acquisition

Data recording in this experiment was performed using SynAmps-2 amplifier and the Neuroscan acquisition software. We recorded EEG data from 64 Ag/AgCl electrodes with electrode positions distributed according to the International 10–20 system. Two unipolar electrodes were placed on the left and right mastoid (M1, M2), and two pairs of bipolar electrodes were also used for horizontal and vertical electrooculography (EOG) recordings to detect eye movements and blinks. The 64-channel EEG data used in the experiment covered the entire head. The EEG and EOG signals were kept under 10 kΩ impedance throughout the experiment, and the sampling rate was set to 1,000 Hz.

To measure the onset moment of movement execution, two electromyography (EMG) electrodes were placed on the deltoid midbundle muscle of the right arm of the subject. Prior to electrode placement, the subject’s skin was gently scrubbed with a skin scrub and then cleaned with an alcohol pad to reduce impedance between the electrodes and the skin. Bipolar Ag/AgCl circular electrodes with a diameter of 2 mm were then placed 2 cm apart in the middle of the deltoid muscle of the subject’s right arm. We synchronous recorded EMG data with EEG data. The impedance of the EMG electrodes was kept below 10 kΩ, and the sampling rate was 1,000 Hz.

### 2.3 Experimental procedure

**Figure 1:**
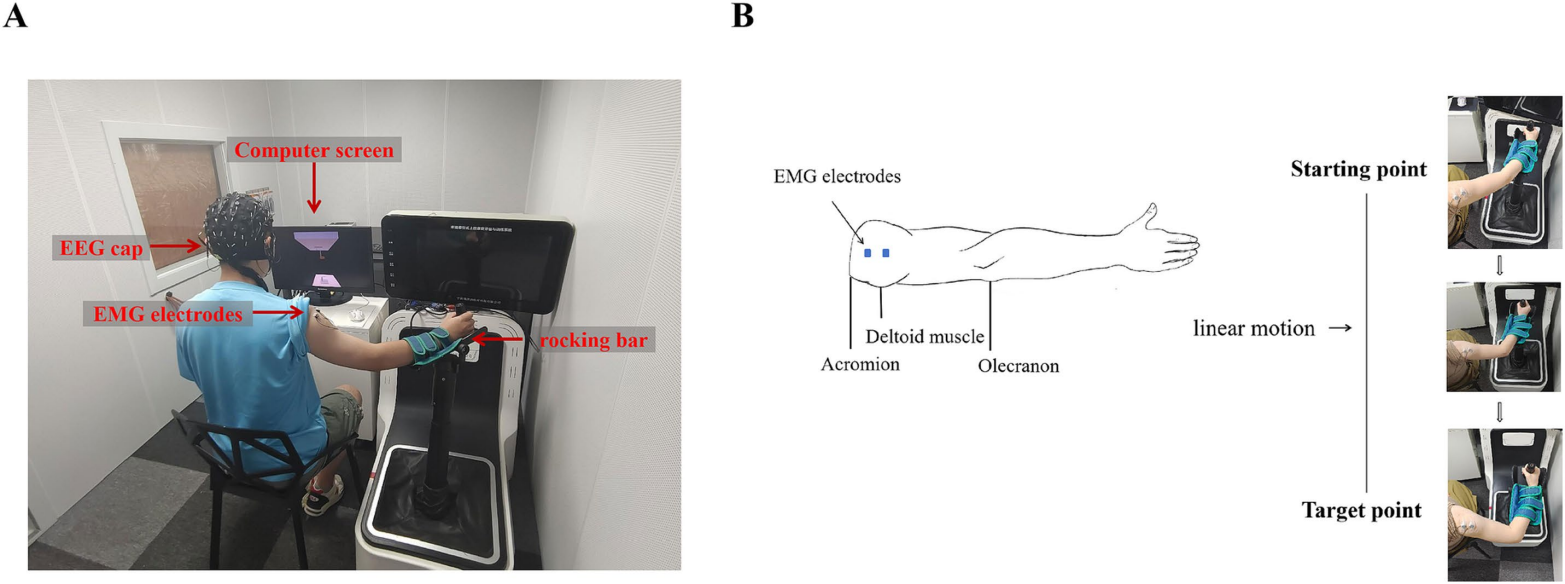
Example of device placement and movement process. **(A)** EEG and EMG data acquisition system. **(B)** Movement process. The left side shows the position of the EMG electrode, and the right side shows the movement trajectory and process.

The whole experiment was conducted in a shielded chamber, which provides insulation from electromagnetic signals and background noise interference, ensuring accurate results. Subjects were seated at a table, with their right hand gripping the rocking bar. The experimental paradigm was displayed on a screen positioned on the table, as shown in Figure 1A. The paradigm was designed by Unity (Unity Technology, San Francisco, USA) software and contained a goal-directed reaching task.

In this experimental paradigm, we designed a spring with upper and lower ends. The upper end of the spring was fixed to the top plate of the scene and its hook, and the lower end of the spring was grasped by the virtual hand. Below the virtual hand, there was a desk and a target hook, as shown in Figure 1A. Subjects were required to focus their attention on the spring, the virtual hand, and the target hook. They manipulated the rocking bar to move the virtual hand and then hung the lower end of the spring, which was grasped by the virtual hand, on the target hook. In order to make the spring reach the target hook, subjects had to perform an elbow flexion maneuver, and the trajectory of the task was a straight line, as shown in Figure 1B.

**Figure 2:**
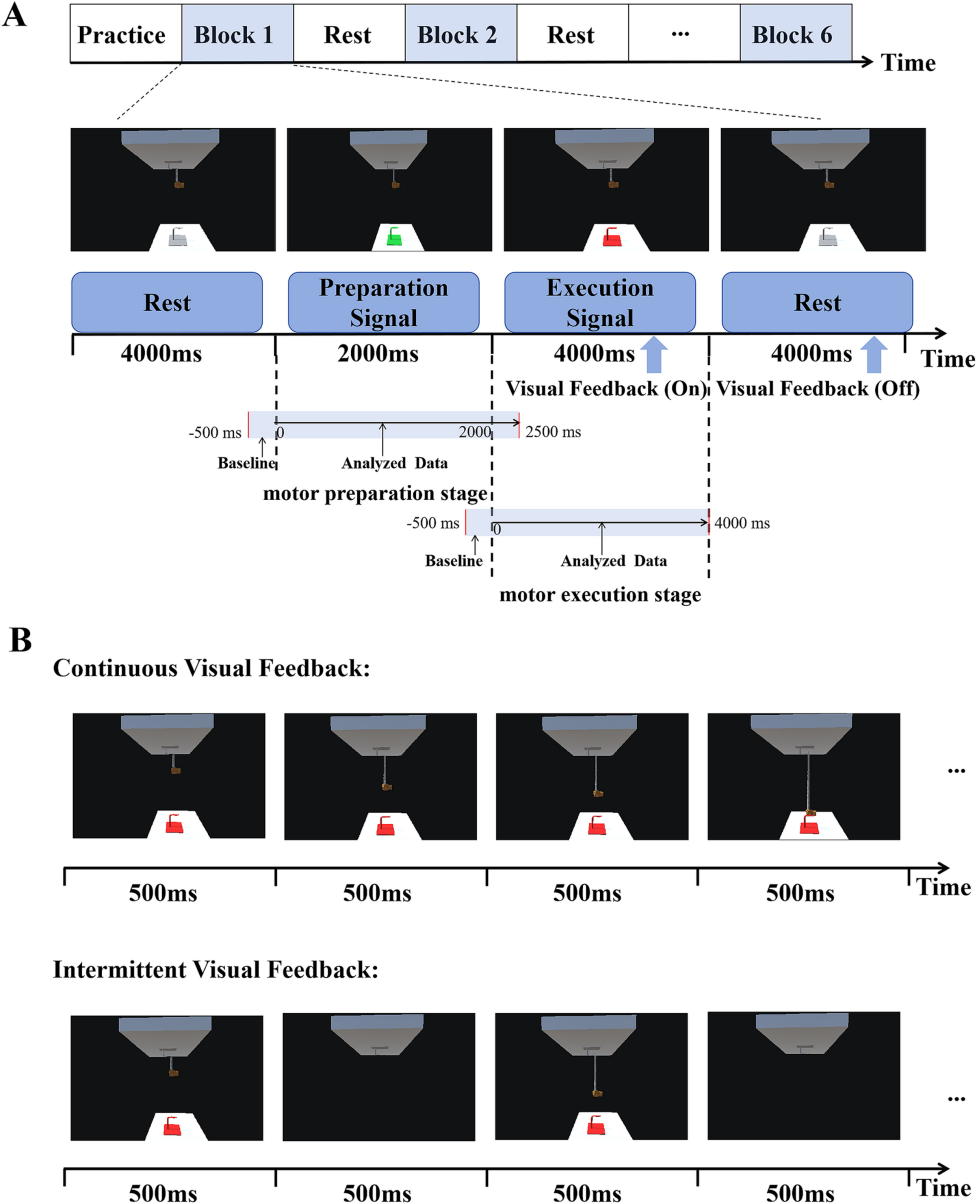
Experimental design. **(A)** Timeline of each experimental trial. The three states displayed include rest (4,000 ms), motor preparation (2000 ms), and motor execution (4,000 ms). Visual feedback was turned on during motor execution and off during rest. **(B)** Two kinds of visual feedback. In the continuous visual feedback condition, subjects could see the target continuously as well as observed changes in the position of the lower end of the spring and the virtual hand. In intermittent visual feedback condition, the target hook, the spring, and the virtual hand appeared and disappeared at a frequency of 1 Hz (500 ms).

There were three periods in this experimental paradigm: rest, preparation, and execution, as shown in Figure 2A. The target hook would have three different colors in the three periods of every trial. The first was the rest period. During this period, the color of the target hook appeared gray for 4,000 ms. The second period was the preparation stage, the color of the target hook changed to green and lasted for 2,000 ms. The target turning green was regarded as the preparation signal for this experiment, indicating the subjects should be prepared for subsequent actions. The last was the execution stage, the color of the target hook changed to red. This served as the task execution signal for the subjects. They were required to immediately control the rocking bar in this stage and successfully hang the virtual spring on the target hook within 4,000 ms. Visual feedback was turned on during execution stage, with changes in the position of the lower end of the spring and the virtual hand were used as visual feedback. That is to say, visual feedback only was on when the target was red and participants were performing tasks. When the virtual spring reached the target hook or the time reached 4,000 ms, it was considered to be the end of a single trial. At this time, the color of the target hook changed from red to gray, and the visual feedback was turned off. The subject returned the rocking bar to its initial position and entered a relaxed rest state.

This paradigm included two different forms of visual feedback: continuous visual feedback (CVF) and intermittent visual feedback (IVF), as shown in Figure 2B. In the continuous visual feedback condition, subjects could see the target continuously as well as observed changes in the position of the lower end of the spring and the virtual hand. In intermittent visual feedback condition, the target hook, the spring, and the virtual hand appeared and disappeared at a frequency of 1 Hz (500 ms), and in order to successfully complete the reaching task, subjects needed to constantly estimate the position of the hook. We did ensure that participants were instructed to maintain a consistent starting arm position between different visual feedback conditions. In both visual feedback conditions, the start and target points in the continuous visual feedback condition were the same as those in the intermittent visual feedback condition, as shown in Figure 1B.

The experiment required subjects to move the rocking bar as smoothly as possible when performing elbow movements, while also avoiding any body movements other than manipulating the rocking bar and to minimize blinking and swallowing. The entire experiment consisted of six consecutive blocks, with three blocks are continuous visual feedback, and the other three blocks are intermittent visual feedback. The first block had continuous visual feedback, followed by the second block with intermittent visual feedback, and so on. Each block contained 35 trials. Before the experiment, there was a practice session to help subjects familiarize themselves with the task rules and the manipulation of the rocking bar. While the subjects were practicing, they were asked to find a comfortable position to manipulate the rocking bar. Before the start of each round of trials, the subjects were asked to adjust their sitting posture and position to ensure consistency with the practice. After each round, subjects were allowed to take a break at their own discretion and in accordance with their physical condition. These breaks lasted for about 5 min or longer if they were fatigued.

### 2.4 Data analysis

EEG data were pre-processed offline using the open-source toolbox EEGLAB (Delorme and Makeig, 2004). Raw EEG data were filtered using a 1–30 Hz bandpass filter and a 50 Hz notch filter. All EEG signals were subsequently re-referenced to the average value of the left and right mastoids (Kornhuber and Deecke, 1965; Nunez et al., 1997). We segmented the EEG data in two stages for analysis and processing, namely, the motor preparation stage and the motor execution stage. In the motor preparation stage, we intercepted the EEG data with a duration of 3,000 ms. The onset of the preparation signal was moment 0 of each epoch, and then the EEG data from 500 ms before the signal to 2,500 ms after the signal was used as the data segment for this stage. In the motor execution stage, EEG data with a duration of 4,500 ms was extracted. The onset of the execution signal was moment 0 of each epoch, and then the EEG data from 500 ms before the signal to 4,000 ms after the signal was used as the data segment for this stage. The first 500 ms of the epochs were used as the baseline to perform baseline correction on EEG data. Finally, Independent Component Analysis (ICA) was used to remove eye movements and motor artifacts.

Data analysis had three levels: behavioral, time domain, time-frequency domain. The results of behavioral analysis are mainly reflected in the subjects’ target accuracy and response time. Target accuracy referred to the proportion of successful target arrivals by the participants within 4,000 ms of motor execution. Response time referred to the interval between the appearance of the execution signal and the subjects’ initiation of action. So the onset time of EMG represented the response time of the subjects. The EMG data was performed with a high-pass filter of 10 Hz to remove movement artifacts. Then, a linear envelope was acquired by using full-wave rectification and a low-pass filter of 30 Hz. The onset of EMG activity for each trial was determined by crossing the threshold at 4 standard deviation (SD) from the mean baseline (Li et al., 2018; Trincado-Alonso et al., 2018; Yilmaz et al., 2013) in either direction. Owing to the concerns associate with the fallibility of identifying the movement onset, the onset of movement was determined by considering the number of consecutive samples (E) where the EMG envelopes exceeded the T. In this study, E was empirically defined as 5. Therefore, when a sequence of more than E (5) consecutive samples surpassed the T, the first time point was assigned as the actual movement onset (Chaisaen et al., 2020).

In the time domain, we analyzed EEG data from various structural and functional areas of the cerebral cortex under different visual feedback conditions. Potential amplitude averaging was performed on EEG data from the frontal-central region (FCz), central region (Cz), and central parietal region (CPz) of the brain. Among them, the FCz electrode is located in the premotor cortex, which is related to motor preparation. The Cz electrode is located in the primary motor cortex, which is associated with motor execution. The CPz is located in the somatosensory cortex, which is associated with sensory perception. The overall mean ERP value was calculated by averaging the EEG data across all subjects in both the continuous and intermittent visual feedback conditions during the movement preparation stage. By observing the average ERP waveforms, we defined MRCP amplitude as the average amplitude of FCz, Cz, and CPz in the period of 1,600-2000 ms.

In the time-frequency domain, to obtain the power spectrum of the EEG signals compared to the baseline, event-related spectral perturbation (ERSP) analysis was carried out by time-frequency decomposition (TFD) using the short-time Fourier transform (STFT) with a fixed Hanning window of 200 ms. In the movement preparation stage of each trial, the preparation signal was taken as moment 0, the time-frequency estimate at each point of the time plane was computed from −500 to 2,500 ms in the time domain and from 1 to 30 Hz in the frequency domain. In the movement execution stage of each trial, which differs from the movement preparation stage by taking the execution signal as moment 0, we intercepted the data from the −500 to 4,000 ms time period for calculation. The calculation method is as follows:


$$P\left(t,f\right)={\left|F\left(t,f\right)\right|}^{2}$$

For each trial, the baseline interval was set from −500 to 0 ms. The subtraction method in the time-frequency domain was used to achieve baseline correction:


$$P_{bl}\left(t,f\right)=P\left(t,f\right)-\bar{R}\left(f\right)$$

Where $\bar{R\;}\left(f\right)$ was the average power spectral density of the baseline interval at each frequency. $P_{bl}\left(t,f\right)$ < 0 was regarded as representing ERD, and $P_{bl}\left(t,f\right)$ > 0 was regarded as representing ERS. The ERD/ERS values in the mu (8 ~ 13 Hz) and beta (14 ~ 30 Hz) at C3, Cz, and C4 were selected for analysis.

### 2.5 Statistical analysis

The data were analyzed and compared using statistical analysis software SPSS (version 26). This is because the previous data processing procedures, including re-referencing, may ameliorate the effects brought about by volumetric conduction, but do not completely solve the problem (Yao, 2001; Yang et al., 2017). Therefore, when performing the statistical analysis, we tested the experimental data for homoscedasticity to ensure that the variance of the data was equal across groups. Specifically, we used Levene’s Test for Equality of Variances to assess the effect of different inter-electrode volume conduction problems on the data. The results of the test showed an *F*-value of 0.002 and a *p*-value of 0.961. This result indicates that the signals between different electrodes had small covariances, and the volume conduction problem did not significantly affect the variance of the data between different electrodes (Ruppert and Aldershof, 1989; Chang et al., 2017).

Then we performed a subsequent statistical analysis of the data. First, we used paired t-test to analyze the results of behavioral data at the different feedback conditions (Continuous, Intermittent). Next, we statistically analyzed the EEG data in the time domain and time-frequency domain, which were divided into two stages: the motor preparation stage and the motor execution stage. In the motor preparation stage, repeated-measures ANOVAs with factors of feedback condition (Continuous, Intermittent) and analysis electrode (ROI, FCz, Cz, CPz) as repeated factors, were used to analyze MRCP results. The ROI was the superimposed mean of the EEG data from the three brain regions FCz, Cz and CPz. And repeated-measures ANOVAs with factors of feedback condition (Continuous, Intermittent) and analysis electrode (C3, Cz, C4) as repeated factors, were conducted to analyze mu-ERD results. In the motor execution stage, in addition to repeated-measures ANOVAs for the mu-ERD results, repeated-measures ANOVAs were also conducted to analyze β-ERD results with factors of feedback condition (Continuous, Intermittent) and analysis electrode (C3, Cz, C4) as repeated factors. The level of significance was judged by the *p*-value, and *p* < 0.05 was considered statistically significant.

## 3 Results

### 3.1 Behavior

**Figure 3:**
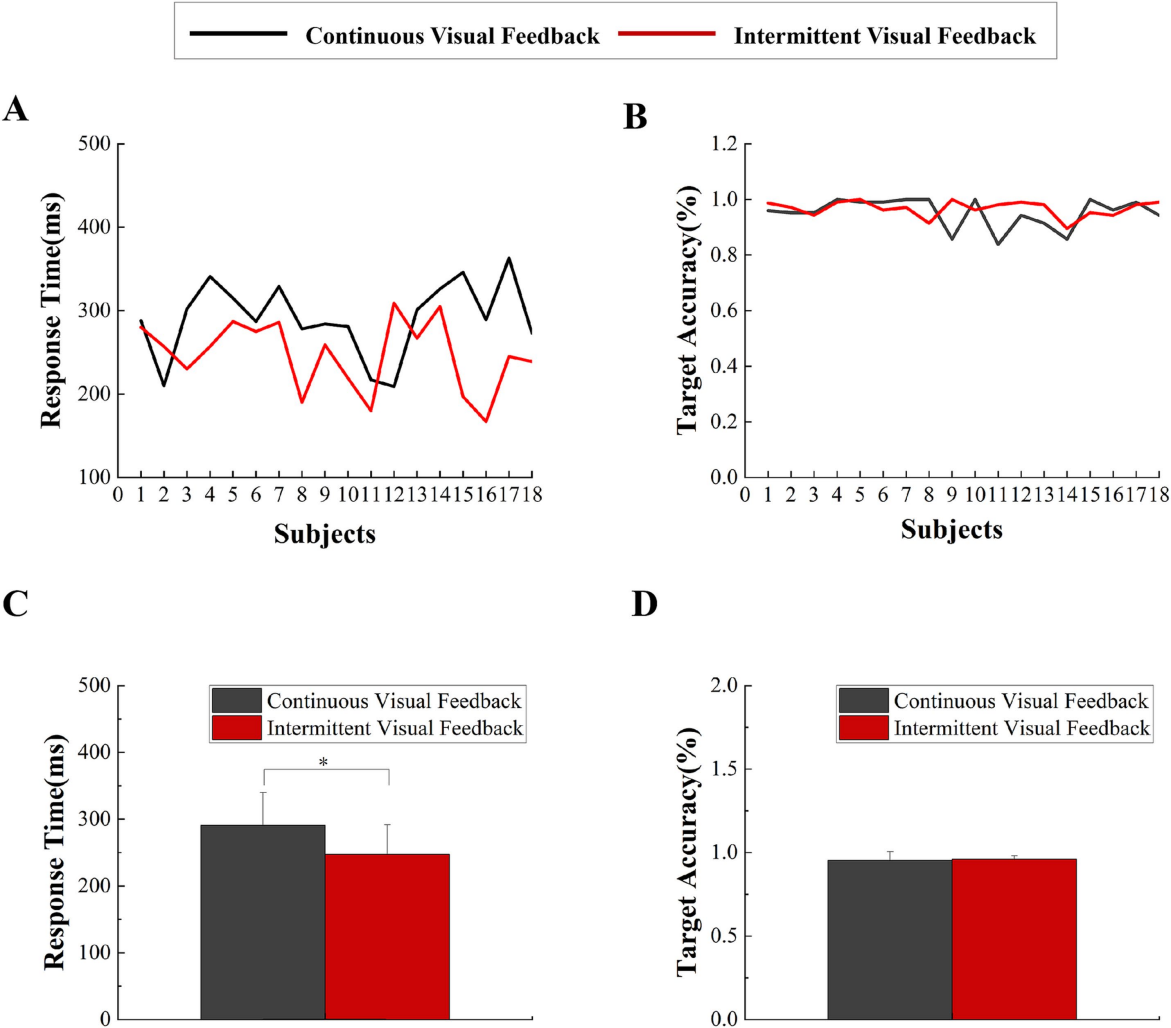
Behavioral data. **(A)** Response times were averaged across all trials for each subject in two visual feedback conditions. **(B)** Target accuracy were averaged across all trials for each subject in the two visual feedback conditions. **(C)** Statistical comparison of response times under continuous visual feedback and intermittent visual feedback. Error bars represent the SD. Significant differences are indicated by asterisks. *p < 0.05. **(D)** Statistical comparison of target accuracy under continuous visual feedback and intermittent visual feedback. Error bars represent the SD. Significant differences are indicated by asterisks. *p < 0.05.

The paired t-tests were conducted to compare the target accuracy and response time between continuous visual feedback and intermittent visual feedback. The subjects had higher accuracy in the intermittent visual feedback condition (Mean ± SD = 0.97 ± 0.02) compared to the continuous visual feedback condition (Mean ± SD = 0.95 ± 0.05). The result of target accuracy was not significant (t = −1.035, *p* = 0.315, Cohen’s d effect size = 0.36). Additionally, the subjects had faster response times in the intermittent visual feedback condition (Mean ± SD = 247 ± 45) compared to the continuous visual feedback condition (Mean ± SD = 291 ± 49). The result revealed a significant statistical difference (t = 3.135, *p* = 0.006, Cohen’s d effect size = 0 0.7; Figure 3). On this basis, the averaged linear envelope results for the continuous and intermittent visual feedback conditions were further averaged to obtain the total linear envelope results. The onset time of the total averaged envelope was approximately 269 ms after the execution signal, which was considered as the start time of the task executed by the subjects.

### 3.2 MRCP

**Figure 4:**
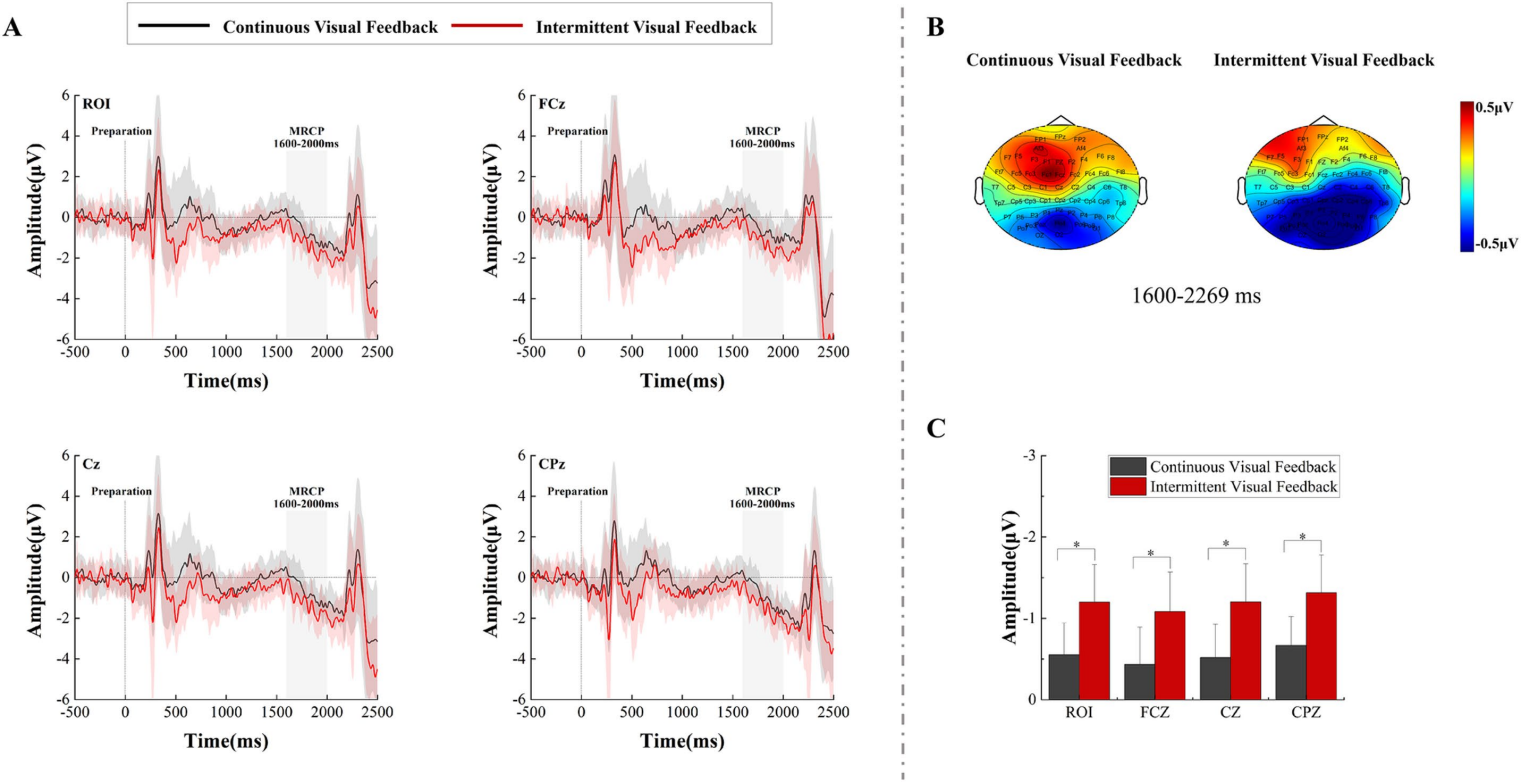
The amplitude differences over recorded brain regions and statistical results under the continuous visual feedback versus intermittent visual feedback. **(A)** The grand average ERP waveform for continuous visual feedback (black solid lines) and intermittent visual feedback (red solid lines) at electrodes FCz, Cz, CPz, and ROI. The MRCP appeared in both visual feedback conditions. Shaded regions represent the SD. **(B)** The brain topographic maps of the late MRCP component. **(C)** Statistical comparison of MRCP amplitude under continuous visual feedback and intermittent visual feedback. Error bars represent the SD. Significant differences are indicated by asterisks.

Time-domain waveforms analyses were conducted on the premotor cortex (FCz), the primary motor cortex (Cz), the somatosensory cortex (CPz), and the region of interest (ROI) during the motor preparation stage. As seen from the time-domain waveforms and topographic maps in Figure 4, different types of visual feedback modulated the amplitude of the MRCP during the movement preparation stage, and the negative trend of the MRCP appeared approximately 1,500 ms after the onset of the preparation signal. A two-factor repeated measures ANOVAs with factors of feedback condition (Continuous, Intermittent) and analysis electrode (ROI, FCz, Cz, CPz) was used to analyze MRCP results (from 1,600 to 2000 ms). The statistical analysis results are shown in Figure 4C. The interaction effect was not significant. However, the main effect of both feedback condition (*F* = 21.283, *p* < 0.001, η^2^ = 0.385, df = 1, SS = 15.454 μV, MS = 15.454 μV) and analysis electrode (*F* = 23.441, *p* < 0.001, η^2^ = 0.408, df = 1.630, SS = 0.979 μV, MS = 0.601 μV) was significant. Post-hoc test analysis showed that regardless of analysis electrode feedback condition, the MRCP amplitude of subjects at CPz electrode was more negative compared to other electrodes (ROI: p < 0.001, SE = 0.024 μV, df = 17; FCz: *p* < 0.001, SE = 0.041 μV, df = 17; Cz: *p* < 0.001, SE = 0.026 μV, df = 17).

### 3.3 ERD/ERS

**Figure 5:**
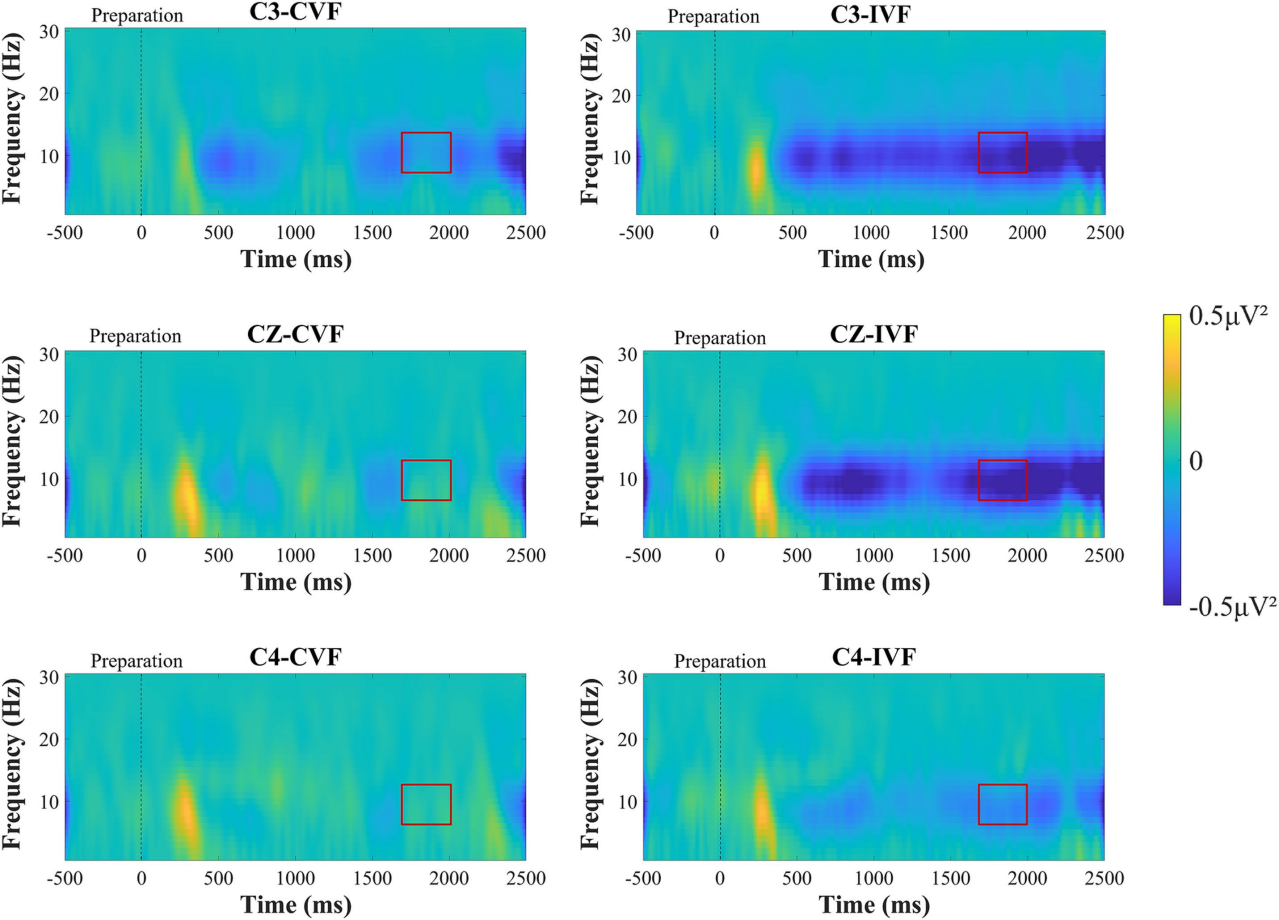
Time-frequency plots recorded over brain regions at the C3, Cz, and C4 electrodes during the motor preparation stage under continuous visual feedback (CVF) and intermittent visual feedback (IVF) conditions. Yellow areas in the time-frequency diagrams indicate the occurrence of ERS, and blue areas indicate ERD phenomena. And the red boxes on the ERD/ERS plot represent the time period required to generate the average. The ERD phenomenon was most evident at the C3 electrode over contralateral motor cortex. Compared with continuous visual feedback, the ERD phenomenon elicited by intermittent visual feedback was more evident.

**Figure 6:**
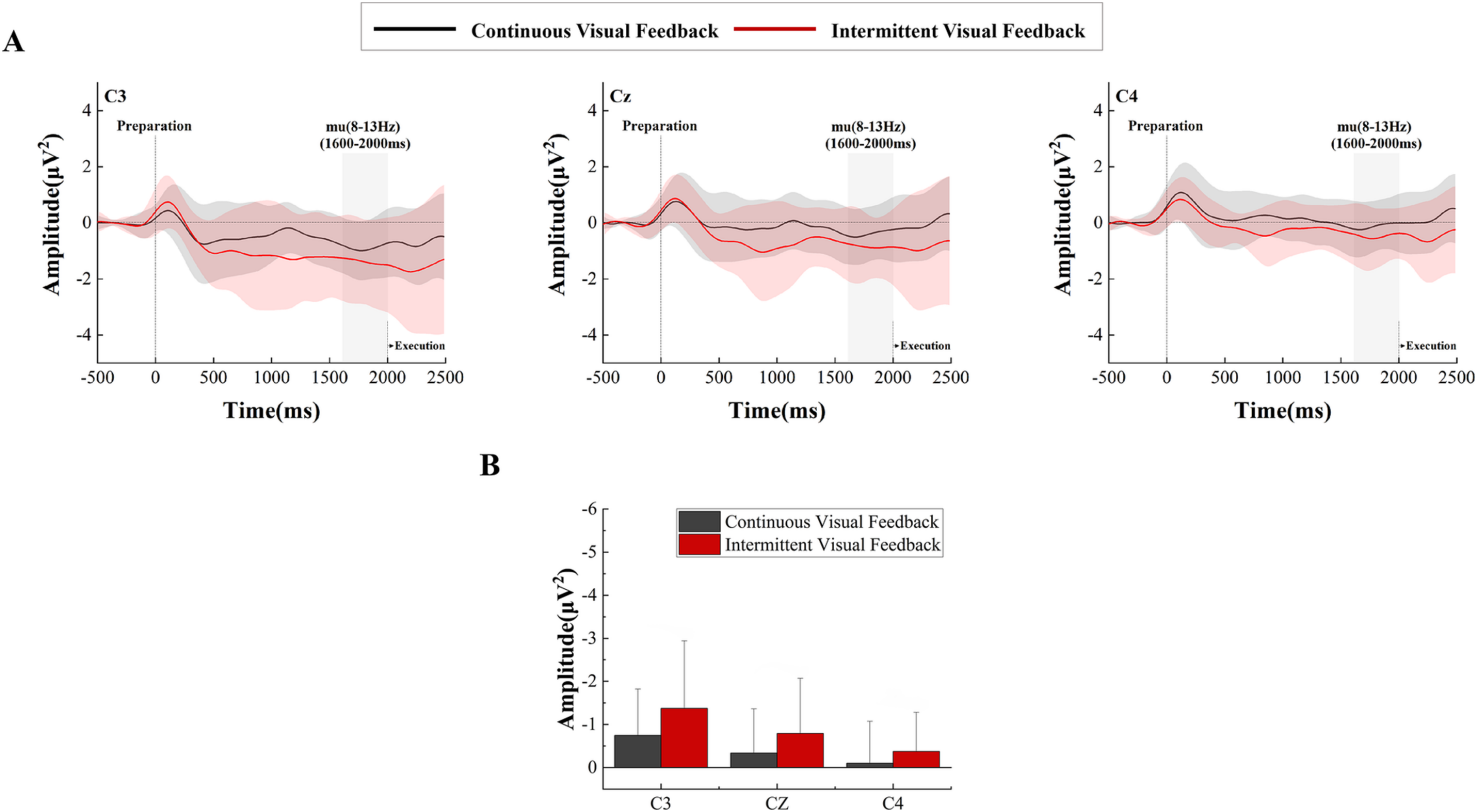
Time-frequency curves and statistical results of the movement preparation stage under continuous versus intermittent visual feedback conditions. **(A)** Time–frequency curves in the mu frequency band for brain regions recorded at the C3, Cz, and C4 electrodes during the movement preparation stage under continuous visual feedback (black solid lines) and intermittent visual feedback (red solid lines) conditions. Transition processes from ERS to ERD in terms of power changes can be observed among the mu frequency band. Shaded regions represent the SD. **(B)** Statistical differences in mu-ERD between the continuous and intermittent visual feedback. Error bars represent the SD.

Figure 5 shows the time-frequency plots calculated from the total average EEG data of all subjects during the motor preparation stage. After 500 ms, the ERD phenomenon in the mu frequency band can be observed at electrodes C3, Cz, and C4. A two-factor repeated measures ANOVAs with factors of feedback condition (Continuous, Intermittent) and analysis electrode (C3, Cz, C4) was used to analyze the power values of the mu frequency band (8-13 Hz). The statistical analysis results are shown in Figure 6B. The interaction effect was not significant, and the main effect of feedback condition (*F* = 1.530, *p* = 0.225, η^2^ = 0.043, df = 1, SS = 5.508 μV, MS = 5.508 μV) was not significant. But from Figure 5 we can observe that the ERD phenomenon in the mu frequency band was more pronounced during intermittent visual feedback compared to continuous visual feedback. In addition, the statistical results showed a significant main effect of electrodes (*F* = 27.691, *p* < 0.001, η^2^ = 0.449, df = 1.261, SS = 12.292 μV, MS = 9.748 μV). Post-hoc test analysis showed that the mu-band power of C3 electrode was larger than that of Cz (*p* < 0.001, SE = 0.071 μV, df = 17) and C4 (*p* < 0.001, SE = 0.146 μV, df = 17) electrodes. As shown in Figure 5, in the continuous visual feedback condition, the obvious mu-ERD phenomenon appeared at electrode C3, while it was weaker at electrode Cz and electrode C4. Under the condition of intermittent visual feedback, the mu-ERD phenomena were clearly observed at the C3 and Cz electrodes. To be specific, under continuous and intermittent visual feedback conditions, the most pronounced ERD phenomenon occurred at the C3 electrode in the contralateral motor cortex.

In addition to this, we analyzed the power changes at the three electrodes (C3, Cz, and C4) in the mu frequency band during the motor preparation stage. As shown in Figure 6A, we observed the power transition process from ERS to ERD in the mu frequency band under continuous visual feedback and intermittent visual feedback conditions. The mu-ERD phenomenon existed in the primary motor cortex (C3, Cz, C4) region 1,500 ms before motor execution, and it was more obvious in the intermittent visual feedback condition. Among the three electrodes (C3, Cz, and C4), mu-ERD exhibited the highest power at the C3 electrode and the lowest power at the C4 electrode.

**Figure 7:**
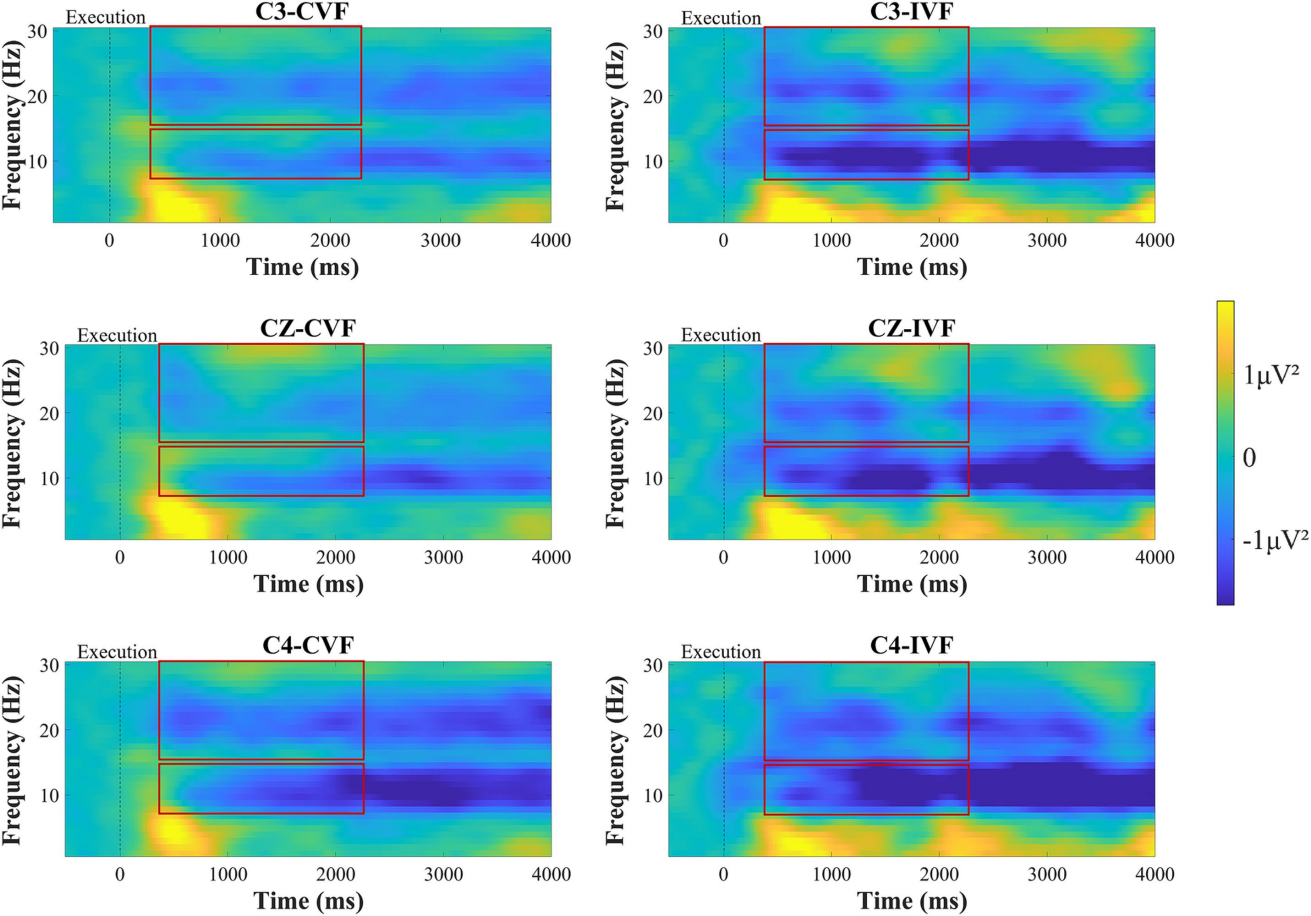
Time-frequency plots of amplitude recorded over brain regions at the C3, Cz, and C4 electrodes during the motor execution stage under continuous visual feedback (CVF) and intermittent visual feedback (IVF) conditions. Yellow areas in the time-frequency diagrams indicate the occurrence of ERS, and blue areas indicate ERD phenomena. The red boxes on the ERD/ERS plot represent the time period required to generate the average.

**Figure 8:**
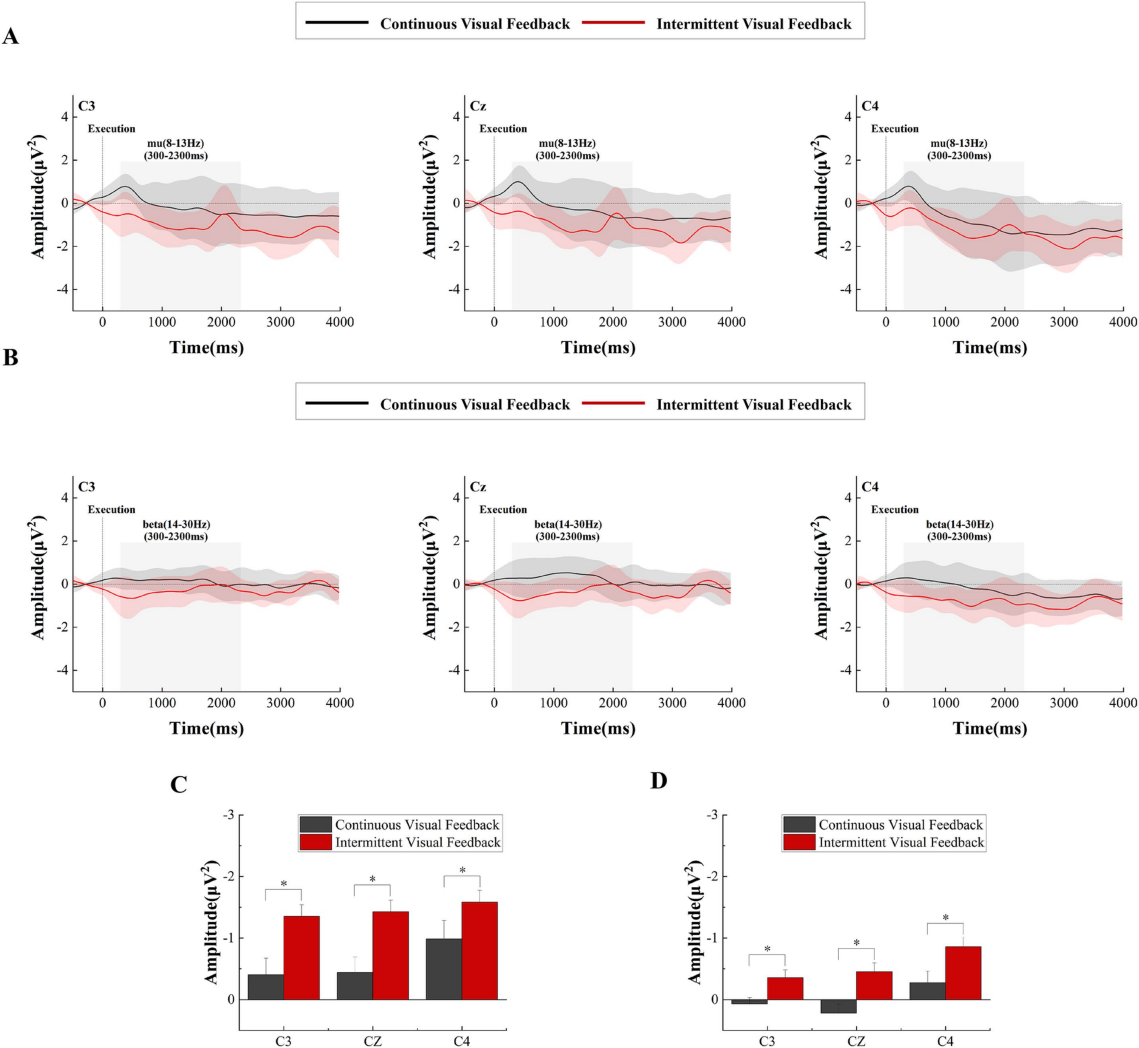
Time-frequency curves and statistical results of the execution stage under continuous versus intermittent visual feedback conditions. **(A)** Time-frequency curves in the mu-band for brain regions recorded at the C3, Cz, and C4 electrodes during the motor execution stage after averaging across all subjects under the continuous visual feedback (black solid line) and intermittent visual feedback (red solid line) conditions. Shaded regions represent the SD. **(B)** Time–frequency curves in the beta-band for brain regions recorded at the C3, Cz, and C4 electrodes during the motor execution stage after averaging across all subjects under continuous visual feedback (black solid lines) and intermittent visual feedback (red solid lines) conditions. Shaded regions represent the SD. **(C)** Statistical differences in mu-ERD between the continuous and intermittent visual feedback. Significant differences are indicated by asterisks. *p < 0.05. Error bars represent the SD. **(D)** Statistical differences in β-ERD between the continuous and intermittent visual feedback. Significant differences are indicated by asterisks. *p < 0.05. Error bars represent the SD.

Figure 7 shows the time-frequency plots of the subjects at the C3, Cz and C4 electrodes during the movement execution stage. Approximately 300 ms after the execution signal, we could clearly observe from the time-frequency maps that there were ERD phenomena in the EEG data at the C3, Cz, and C4 electrodes in the primary motor cortex. A two-factor repeated measures ANOVAs with factors of feedback condition (Continuous, Intermittent) and analysis electrode (C3, Cz, C4) as repeated factors, was used to analyze the power values of the mu frequency (8-13 Hz) and beta frequency (14–30 Hz) bands, respectively. The statistical analysis results are shown in Figures 8C,D. For the mu frequency band (8-13 Hz), the interaction effect was not significant. The main effect of feedback condition (*F* = 6.988, *p* = 0.012, η^2^ = 0.170, df = 1, SS = 19.210 μV, MS = 19.210 μV) and electrodes (*F* = 14.058, *p* < 0.001, η^2^ = 0.293, df = 1.453, SS = 3.520 μV, MS = 2.423 μV) was statistically significant. Post-hoc test analysis showed that the mu-ERD phenomenon was more pronounced at the C4 electrode than at the C3 (*p* = 0.001, SE = 0.104 μV, df = 17) and Cz (*p* < 0.001, SE = 0.081 μV, df = 17) electrodes in the ipsilateral motor cortex during motor execution stage. For the beta frequency band (14-30 Hz), two-way repeated measures analysis of variance showed that the main effect of both feedback condition (*F* = 10.227, *p* = 0.003, η^2^ = 0.231, df = 1, SS = 8.408 μV, MS = 8.408 μV) and analysis electrode (*F* = 14.511, *p* < 0.001, η^2^ = 0.299, df = 1.434, SS = 4.602 μV, MS = 3.211 μV) was significant. Same as the mu-ERD, post-hoc test analysis showed that the beta-ERD phenomenon was more obvious at the C4 electrode than at the C3 (*p* = 0.004, SE = 0.12 μV, df = 17) and Cz (*p* < 0.001, SE = 0.08 μV, df = 17) electrodes in the ipsilateral motor cortex.

**Figure 9:**
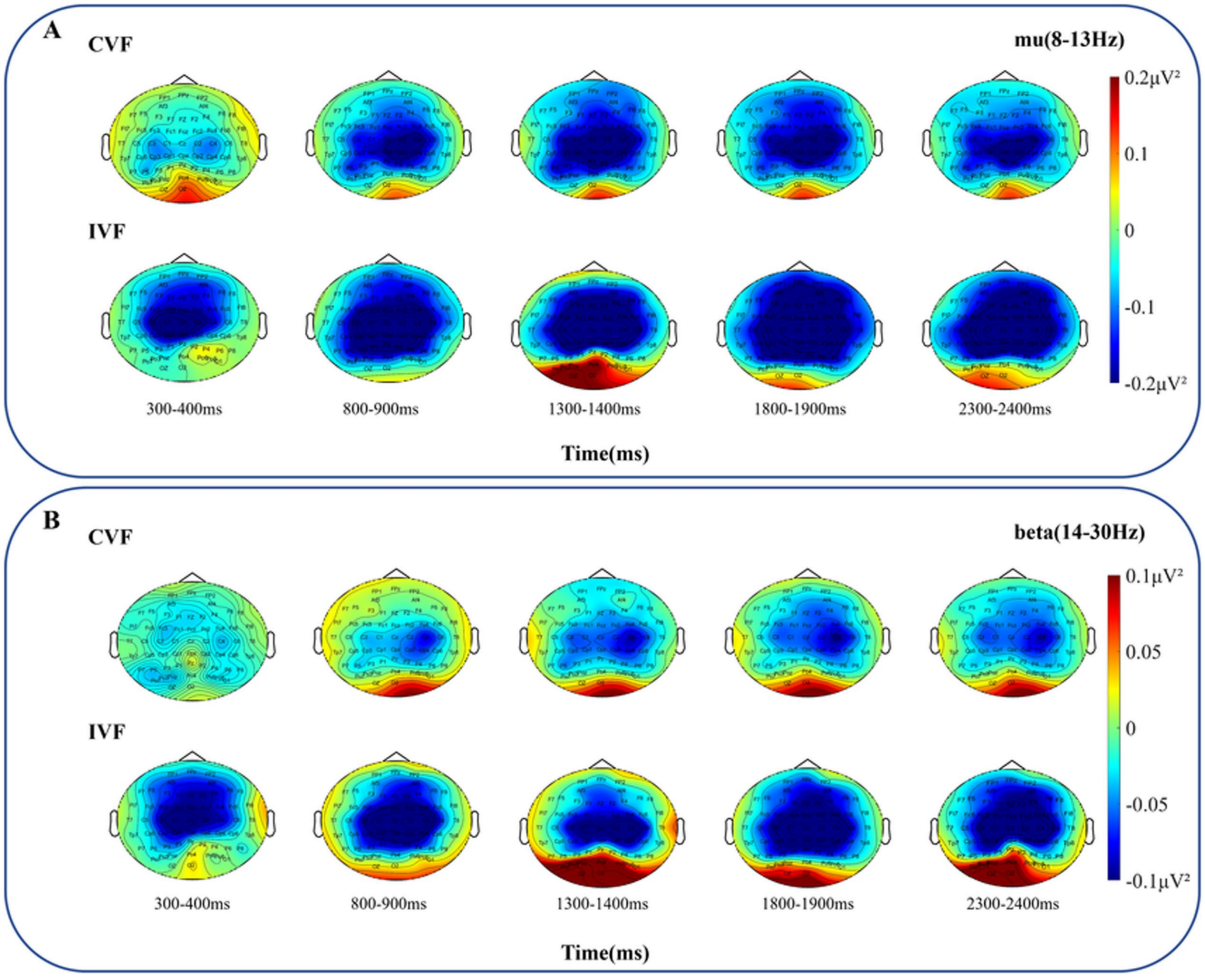
The grand average topography of spectral characteristics in the mu and beta frequency bands was analyzed for all subjects during the motor execution stage, under both continuous visual feedback (CVF) and intermittent visual feedback (IVF) conditions. **(A)** The brain topographic maps in the mu frequency band. **(B)** The brain topographic maps in the beta frequency band. The ERD/ERS process at all electrodes of entire brain regions in response to the continuous visual feedback and intermittent visual feedback is shown.

Beyond that, we analyzed the power changes in the mu and beta frequency bands in the primary motor cortex, as shown in Figures 8A,B, respectively. Overall, the power of the mu and beta frequency bands was greater in the intermittent visual feedback condition. However, there was an upward and downward trend change in the power of the mu and beta frequency bands under the intermittent visual condition, as shown in Figure 8A where the power in the mu and beta bands rose at the moment of 2000 ms, which could be due to the visual stimuli induced by the target, the spring, and the virtual hand. Figures 9A,B show the brain spectral feature maps in the mu and beta frequency bands during the motor execution stage, respectively. ERD/ERS was shown at all electrodes in all brain regions under both continuous and intermittent visual feedback conditions. According to the EMG analysis, the subjects started the task 269 ms after receiving the execution signal. Therefore, the spectrally characterized brain topography maps were plotted starting from 300 ms after execution signal. From the Figure 9A, we could find that in the continuous visual feedback condition, mu-ERD of the primary motor cortex dominated in the 300–400 ms time period. As subjects performed the task, the region of mu-ERD became more pronounced. The mu-ERD appeared in the frontal region during the 800 ms-900 ms time period, and its range further expanded to the prefrontal region during the 1,300 ms-1400 ms time period. In the intermittent visual feedback condition, the prefrontal, frontal, and central regions showed the mu-ERD phenomenon at the early stage of motor execution. The mu-ERD was particularly evident in the prefrontal, frontal, and central regions during the 1800 ms-1900 ms time period. Compared to continuous visual feedback, mu-ERD was significantly stronger under intermittent visual feedback during the motor execution stage. The mu-ERS appeared significantly in the occipital region during 1,300–1400 ms and 2,300–2400 ms. This might be due to visual stimulation induced by intermittent visual feedback. From Figure 9B, it could be noted that the process of changes in the spectral features of the beta frequency band in brain topographic map was roughly similar to that of the mu frequency band. It was noteworthy that beta-ERD could be found to be more pronounced in the ipsilateral motor cortex during the 800–900 ms time period under the continuous visual feedback condition.

## 4 Discussion

In the present study, we recorded and analyzed changes in brain activity of subjects during the motor preparation and execution stages in a goal-directed reaching task under continuous and intermittent visual feedback conditions. The findings showed that target accuracy was higher under intermittent visual feedback compared to continuous visual feedback, and the response time was faster than that under continuous visual feedback. Subjects evoked larger MRCP amplitudes and more intense ERD in the intermittent visual feedback condition.

### 4.1 Behavior

Firstly, compared to continuous visual feedback, the target accuracy was higher under intermittent visual feedback conditions. Consistent with previous research, it also highlights the crucial role of visual feedback in motor control and learning (Goodbody and Wolpert, 1999; Wolpert et al., 2003). Secondly, under intermittent visual feedback conditions, the response time of subjects was faster. This may be because intermittent feedback increased the subjects’ concentration and alertness (Ballester et al., 2017; Jones et al., 2010), so that they were able to respond to the execution signal more quickly.

### 4.2 MRCP

From the ERP analysis results of motor preparation in Figure 4A, continuous and intermittent visual feedback regulated different MRCP amplitudes. Around 600 ms before the execution of the movement, the amplitude of MRCP in subjects was larger in the intermittent visual feedback condition, and the MRCP amplitude was largest at the CPz above the somatosensory cortex. MRCP is an EEG index used to study motor preparation within the brain, and its negative wave amplitude correlates with motor preparation and also reflects the level of excitation in the motor cortex of the brain (Elbert and Rockstroh, 1987; Birbaumer et al., 1990). This suggests that under intermittent visual feedback condition, subjects had increased cortical excitability and cortical activation. In addition, some researchers have correlated the negative amplitude of MRCP with the effort required (Frömer et al., 2012; Tomyta and Seki, 2020; Berchicci et al., 2017; Jochumsen et al., 2017; Wright et al., 2011). Our findings support this view. Compared to continuous visual feedback, the goal-directed reaching task in the intermittent visual feedback condition may be considered a more complex task, because it required subjects to more accurately adjust their motor plans to accommodate the intermittent nature of the visual feedback. That is, the task in the intermittent visual feedback condition required subjects to put more effort and planning into the upcoming movement.

A large number of studies have shown that MRCP peak potentials usually occur at Cz or FCz electrodes (Dirnberger et al., 2000; Li et al., 2018). Our study found that the MRCP amplitude was more negative at the CPz electrode than at the Cz and FCz electrodes. It is reasonable to assume that intermittent visual feedback strengthens the sensory loop between motor intention and somatosensory feedback. Motor commands during motor preparation must be planned based on sensory information obtained in the past. That is, the next motor preparation process is influenced by the previous one motor execution process. During intermittent visual feedback, subjects would have a visuomotor correction in the process of reaching a goal (Vaillancourt et al., 2006). It is possible that this motor correction process results in subjects experiencing different sensory information, which in turn leads to increased motor anticipation and planning during motor preparation. During continuous visual feedback, due to the uninterrupted provision of visual stimuli, frequent corrections by the brain may not be necessary as the visual information is consistently updated in real time. The continuous and immediate availability of visual information diminishes the requirement for specific moments of visuomotor corrections (Körding and Wolpert, 2004). Moreover, intermittent visual feedback might involve memory processes. It had been demonstrated that additional cognitive loads, such as attention and memory processes, could promote localized cortical discharges under the frontal electrodes of the dorsolateral prefrontal cortex (DLPFC), which also increased the amplitude of MRCP (Dirnberger et al., 2000) and enhanced the brain activations.

### 4.3 ERD/ERS

During the motor preparation stage, we observed more pronounced mu-ERD in the intermittent visual feedback condition than in the continuous visual feedback condition (Figure 5). Mu-ERD is generally regarded as a reliable correlate of increasing cellular excitability in thalamocortical systems during cortical information processing (Middendorf et al., 2000) and is observed in motor observation, imagery, preparation, and execution stages (Neuper et al., 2006). Our study showed that intermittent visual feedback increased brain activation as well as cortical excitability in subjects compared to continuous visual feedback. It has been shown that mu-ERD appear in the motor cortex contralateral to the moving limb during the movement preparation phase, and this was again verified by our findings. The most significant mu-ERD feature was presented at the C3 electrode.

As the movement is executed the amplitude of mu-ERD decreases and spreads to bilateral motor cortex. In addition to mu-ERD, β-ERD also occurs during motor execution (Neuper et al., 2006; li et al., 2018). From Figure 7, we can see that the time-frequency analysis results of motor execution are consistent with motor preparation, and our study found that the mu-ERD and β-ERD were more pronounced in the intermittent visual feedback condition. First, subjects may have evoked stronger proprioceptive control in the intermittent visual feedback condition which led to enhanced activation of motor cortex in the brain (Sober and Sabes, 2003). This is due to the fact that intermittent visual feedback requires the brain to rely more heavily on proprioceptive information to guide and adjust movements, given the gaps in visual information. The brain needed to judge the location of the target when the subjects executed the task in the intermittent visual feedback condition. When the virtual hand and the spring disappears, subjects needed to constantly estimate the target position and moved the rocking bar, which induce the control of proprioceptive integration. Training related to proprioceptive integration in goal-directed tasks strongly activates the extended motor cortex (including premotor cortex, primary motor cortex and parietal cortex; Kang et al., 2012). In contrast, continuous visual feedback provides real-time updates that may reduce the reliance on proprioceptive control by offering consistent visual guidance. Secondly, intermittent visual feedback causes subjects to make visual corrections while performing a movement task, that is, to adapt to changes in feedback by continuously adjusting their movement strategy. This correction involves both visual actuation and visual recalibration, and Adams et al. (2010) showed that this feedback mechanism prompts the brain to perform motor recalibration, which improves the accuracy of the motor program. And a study by Vaillancourt et al. (2006) also found that intermittent visual feedback increased neural activity in parietal cortex and primary motor cortex, suggesting that the brain’s neural mechanisms for processing and adjusting to motor control are enhanced. Additionally, when the brain detects an error, the visual feedback prompted the subject to concentrate more in the next trial (Zhang et al., 2021). So that the brain became more active during the motor execution stage in the intermittent visual feedback condition. In addition to that, from Figure 7, it was known that both contralateral and ipsilateral motor cortices exhibited ERD, which indicated that involvement of bilateral brain activity in the motor task.

The present study has some limitations. In terms of experimental design, we focused on the characteristic of visual feedback frequency. The difference in accuracy between the visual conditions was not significant, which may be due to a ceiling effect resulting from the task design. Although our design was intended to explore the role of visual feedback in the reaching task, we must recognize that participants were likely to have gained additional information about positional cues through proprioception, which may have mitigated their reliance on visual cues to some extent. This is a limitation that exists in this study. In addition, since our experiment only analyzed visual feedback at one frequency, it is uncertain whether these results can be generalized to other frequencies of visual feedback. In the future, we will continue exploring the impact of different frequencies of visual feedback on motor-related brainwave characteristics in order to gain a more comprehensive understanding of the role of visual feedback in motion control. We hope to find the optimal frequency for visual feedback that can provide theoretical and practical foundations for motion training and rehabilitation.

## 5 Conclusion

In the current study, we used comprehensive EEG analysis methods that integrates event-related potential, brain topography, and time-frequency maps to investigate variations in brain activity during both the motor preparation and execution stages in a goal-directed reaching task. Specifically, we compared brain activity responses under two different visual feedback conditions: continuous versus intermittent visual feedback. EEG characteristics in the time-domain and time-frequency domain were more pronounced in subjects during the reaching task under intermittent visual feedback compared to continuous visual feedback. During the motor preparation stage, the MRCP amplitude was larger in the intermittent visual feedback condition, with the largest MRCP peak being at CPz electrode over the somatosensory cortex. In the time-frequency domain, ERD activation in the mu frequency band was more pronounced of amplitude in the intermittent visual feedback condition, and contralateral motor cortex activations was significantly stronger. During the motor execution stage, both the ERD in the mu frequency band and the beta frequency band were larger in the intermittent visual feedback condition, and both bilateral motor cortices were involved in motor execution. These findings provide robust evidence that intermittent visual feedback enhanced EEG characteristics and induced more prominent cortical activation during the motor preparation and execution stages compared to continuous visual feedback conditions. Therefore, when designing goal-directed reaching tasks, that require positional information as visual feedback, consideration should be given to providing intermittent visual cues of position.

## Data Availability

The original data and materials presented in this article can be obtained from the corresponding authors upon request.
